# Added Value of Dynamic EMG in the Assessment of the Equinus and the Equinovarus Foot Deviation in Stroke Patients and Barriers Limiting Its Usage

**DOI:** 10.3389/fneur.2020.583399

**Published:** 2020-11-19

**Authors:** Isabella Campanini, Michela Cosma, Mario Manca, Andrea Merlo

**Affiliations:** ^1^LAM-Motion Analysis Laboratory, Neuromotor and Rehabilitation Department, S. Sebastiano Hospital, Azienda USL-IRCCS di Reggio Emilia, Reggio Emilia, Italy; ^2^Motion Analysis Laboratory, Department of Neuroscience and Rehabilitation, Azienda Ospedaliero Universitaria di Ferrara, Ferrara, Italy; ^3^Rehabilitation Unit, Fondazione Poliambulanza, Brescia, Italy; ^4^Merlo Bioengineering, Parma, Italy

**Keywords:** dynamic EMG, surface EMG, stroke, gait analysis, rehabilitation, equinus deformity, equinovarus deformity, physiotherapy

## Abstract

Equinus (EFD) and equinovarus foot deviation (EVFD) are the most frequent lower limb deformities in stroke survivors. The equinus component can be triggered by a combination of dorsiflexor deficits, plantar flexor overactivity, muscle stiffness, and contractures. The varus component is typically due to an imbalance between invertor and evertor muscle actions. An improvement in identifying its causes leads to a more targeted treatment. These deformities are typically assessed via a thorough clinical evaluation including the assessment of range of motions, force, spasticity, pain, and observational gait analysis. Diagnostic nerve blocks are also being increasingly used. An advantage of dynamic electromyography (dEMG) is the possibility of measuring muscle activity, overactivity or lack thereof, during specific movements, e.g., activity of both ankle plantar flexors and dorsiflexors during the swing phase of gait. Moreover, fine-wire electrodes can be used to measure the activity of deep muscles, e.g., the tibialis posterior. An impediment to systematic use of dEMG in the assessment of EFD and EVFD, as a complimentary tool to the clinical evaluation, is a lack of evidence of its usefulness. Unfortunately, there are few studies found in literature. In order to fill this void, we studied three pairs of patients suffering from chronic hemiparesis consequent to a stroke, with EFD or EVFD. At the initial evaluation they all displayed the same clinical traits, very similar walking patterns, and an overlapping gait kinematics. However, the patterns of muscle activity differed considerably. dEMG data acquired during walking provided information that was not available from the sole clinical assessment. The contribution of this information to the subsequent clinical and rehabilitation process was discusses along with the barriers that limit the use of dEMG as a routine tool in neurorehabilitation.

## Introduction

Following an upper motor neuron lesion, patients may develop acquired deformities in the lower limbs that impair or inhibit walking. The most frequent lower limb acquired deformities in stroke survivors are the equinus (EFD) and the equinovarus foot deviation (EVFD) (1). They are characterized by a downward deviation and an internal rotation of both the ankle and foot (2, 3). Both are often associated with clawed toes. Ankle dorsiflexors (DF), plantarflexors (PF), invertor, and evertor muscles and toe flexors and extensors control ankle-foot movements and are typically involved with EFD/EVFD in various ways. These deformities are caused by several factors that are found in the different combinations of paresis, overactivity, and altered motor control (paresis, co-contractions, spasticity, dystonia, and other manifestations of muscle overactivity), changes in soft tissues that gradually result in stiffness, contractures and secondary joint limitations due to disuse (4, 5).

The combination of these phenomena is different in each patient and results in joint alterations during gait, which must be evaluated in dynamic conditions because they may not be detectable and measurable by a clinical evaluation. Clinical evaluation and observation of a patient's gait may not be sufficient to establish the causes responsible for the observed deviation and walking alterations. The contribution of gait analysis (GA), and in particular dynamic EMG (dEMG), can be used to discriminate between different causes and thus support the decision-making process in order to formulate the most appropriate therapeutic program (5–9). The presence/absence of EMG activity and the type of activation (e.g., continuous, with bursts) detected (9–15) leads to different choices in terms of focal neuromuscular blocks, non-pharmacological treatments, and neuro-orthopedic surgery.

Failure to use dEMG and GA during the decision-making process could be due to the presence of some barriers (13, 16). In this manuscript we tried to overcome this issue. We will present six patients with very similar gait patterns and different dEMG that provides information not available by the sole clinical assessment.

## Materials and Methods

### Patients and Clinical Assessment

Six subjects with stroke and EFD or EVFD during gait and showing clinical and kinematic overlapping patterns were selected from the authors' databases.

Written informed consent was obtained from the patients for the publication of any potentially identifiable images or data included in this article.

Based on the clinical assessment we extracted the following variables from the patients' records: age, sex, affected side, time elapsed from stroke and its etiology, maximum passive ankle dorsiflexion measured with the knee both extended and flexed, score of the Modified Ashworth Scale at the triceps surae and the soleus and Functional Ambulation Category score (FAC), along with the subsequent rehabilitative choices (17).

### Instrumental Assessment

From the patients' GA we collected lower limb kinematics and dEMG activity. GA was acquired during walking at spontaneous speed on a level surface. At least five strides per subject were considered. Markers for kinematic analysis were placed according to Conventional protocol (18). Surface electrodes (2 cm interelectrode distance) were placed on the lower limb muscles at specific positions, defined as the minimum crosstalk areas (19, 20). Fine wire electrodes were inserted based on the anatomical landmarks in accordance to recommendations by Perotto et al. (21) and further confirmed by ultrasound guidance (22). Their proper placement was then verified by electrical stimulation. One laboratory was equipped with a BTS-Smart system for 3D motion capture and a BTS FreeEmg device (BTS Bioengineering, Italy). The other laboratory was equipped with a Bonita Vicon system (Vicon, United States) and ZeroWire electromyograph (Cometa, Italy). Data were analyzed using the EMG Easy Report software (MerloBioengineering, Parma, Italy). Both laboratories had been previously cross validated for GA (23).

### Interpretation of dEMG

Muscles that could play a role in EFD and EVFD deformities are summarized in Table 1, which shows, for each muscle, the different combinations of paresis (a lack of voluntary motor command), overactivity (such as spasticity, tension-dependent muscle involuntary activation, out of phase muscle activation, and a reduced ability to relax muscles), soft tissue contracture (especially muscle shortening and joint retraction), and increased stiffness (a reduction of muscle elasticity and extensibility) (24) that could result in foot deviation during both the stance and the swing phases.

**Table 1:** dEMG-based interpretation of EFD and EVFD causes.

**Plantarflexor and dorsiflexor muscles**	**EMG pattern (classification) and effect**	
	**Stance phase**	**Swing phase**
Flexor digitorum longus (FDL)	Premature in LR/Prolonged in PSw (overactivity) **E, V**	Out of phase (overactivity) **V**
Flexor hallucis longus (FHL)	Premature in LR/Prolonged in PSw (overactivity) **E, V**	Out of phase (overactivity) **V**
Gastrocnemius medialis (GAM)	Premature in LR/Prolonged in PSw (overactivity) **E** Absent or minimum continuous activity (contracture, stiffness) **E**	Out of phase (overactivity) **E** Minimum continuous activity (stiffness, contracture) **E**
Gastrocnemius lateralis (GAL)	Premature in LR, prolonged in PSw (overactivity) **E** Absent or minimum continuous activity (contracture, stiffness) **E**	Out of phase (overactivity) **E** Minimum continuous activity (stiffness, contracture) **E**
Soleus (SOL)	Premature in LR/Prolonged in PSw (overactivity) **E, V** Absent or minimum continuous activity (contracture, stiffness) **E, V**	Out of phase (overactivity) **E, V** Minimum continuous activity (stiffness, contracture) **E, V**
Tibialis posterior (TP)	Premature in LR/Prolonged in PSw (overactivity) **V** Absent or minimum continuous activity (contracture, stiffness) **V**	Out of phase (overactivity) **V** Minimum continuous activity (stiffness, contracture) **V**
Tibialis anterior (TA)	Prolonged in MSt-TSt (overactivity) **V** Absent in LR and in PSw (weakness) **E, V**	Absent during whole swing (weakness) **E** Absence of the second peak in TSw (weakness) **E, V** Continuous (overactivity without EDL activity) **V**
Extensor digitorum longus (EDL)	Absent in LR and in PSw (weakness) **E, V**	Absent during whole swing (weakness) **E, V**
Extensor hallucis longus (EHL)	Prolonged in MSt-TSt (overactivity) **V** Absent in LR and in PSw (weakness) **E**	Absent during whole swing (weakness) **E** Absence of the second peak in TSw (weakness) **E** Continuous (overactivity) **V**

***E**, Equinus; **V**, Varus; LR, Loading Response, that includes initial contact, MSt, Mid Stance; TSt, Terminal Stance; PSw, Pre-Swing; TSw, Terminal Swing*.

Table 1 was used as reference in the analysis of the cases presented in this study. This was developed by the authors throughout years of clinical and instrumental practice and was also based on available literature (2, 9, 11, 13, 25). For all cases, we discussed the causes of EFD and EVFD based on the dEMG patterns and presented the refinement of the intervention proposal. Finally, the added value of dEMG during walking to clinical decision-making and to physiotherapy has been discussed, along with the barriers that currently limit its use.

## Results

**Figure 1:**
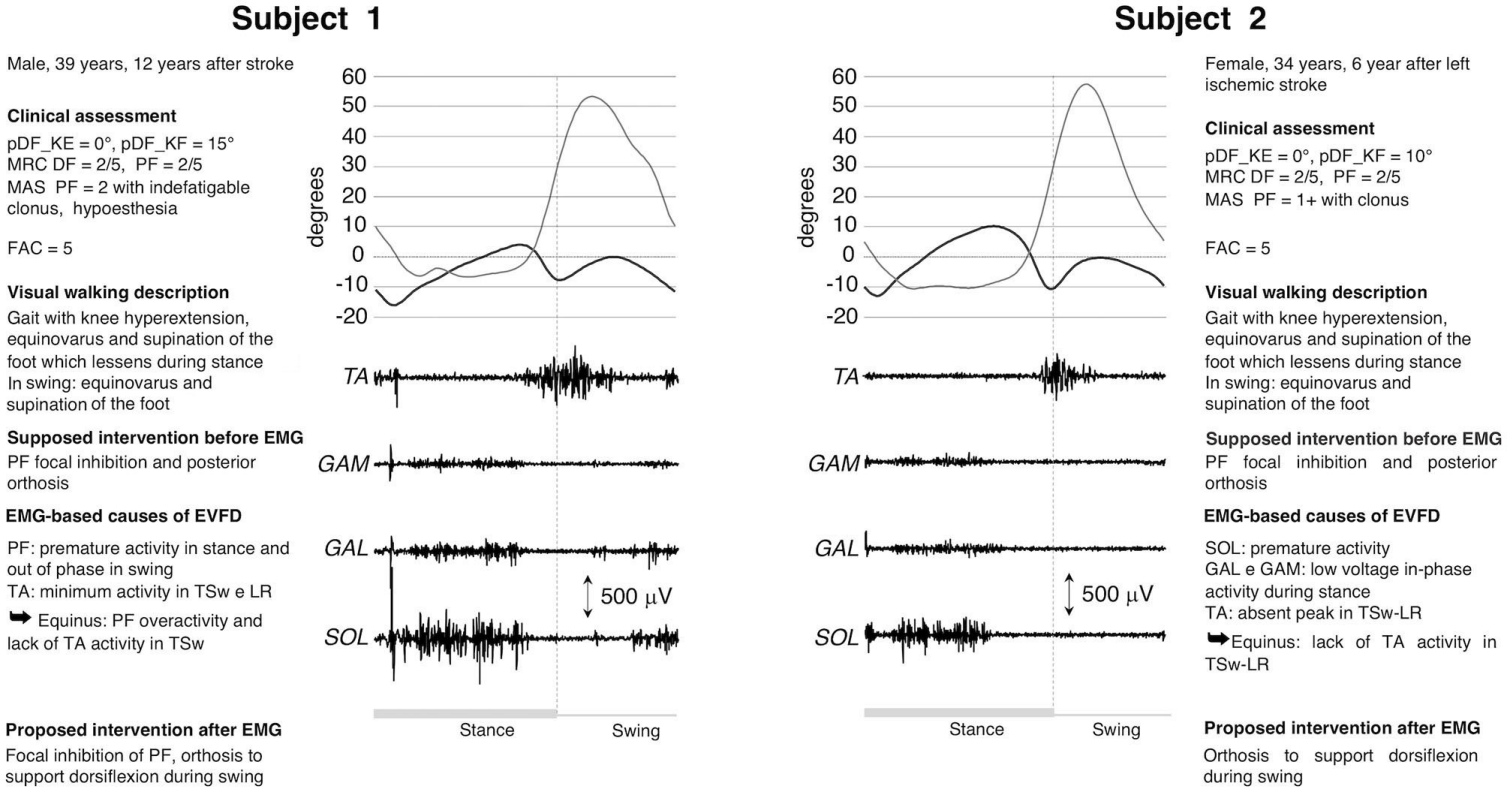
dEMG, knee and ankle kinematic during a gait cycle for two post-stroke patients presenting similar clinical characteristics and very similar kinematics. Clinical assessment included: maximum passive ankle dorsiflexion measured with the knee extended (pDF_KE) and flexed (pDF_KF); plantarflexor (PF) and dorsiflexor (DF) muscles force measured with the Medical Research Council scale (MRC), PF spasticity scored with the Modified Ashworth scale (MAS), and the walking ability measured with the Functional Ambulation Category scale (FAC). See Table 1 for muscle-related acronyms.

Subjects #1 and #2 (Figure 1) exhibited a gait with foot supination and equinus, reducible during stance, and a hyperextended knee in Mid Stance (MSt) and Terminal Stance (TSt). Clinically, both patients had the same slightly reduced passive ankle mobility that is usually compatible with a modest shortening of PF, a marked weakness of DF and PF and a reflex overactivity of PF, the latter more pronounced in Patient 1. Following clinical evaluation and in the absence of significant signs of contracture the treatment hypothesis for both was the use of a posterior orthosis to support the weakness of DF and a generic PF inhibitory focal treatment. However, when dEMG assessment was performed, the two subjects showed different dEMG patterns during swing. In Patient 2, the use of focal inhibition was excluded because of the absence of overactivity during gait, while it was confirmed in Patient 1.

**Figure 2:**
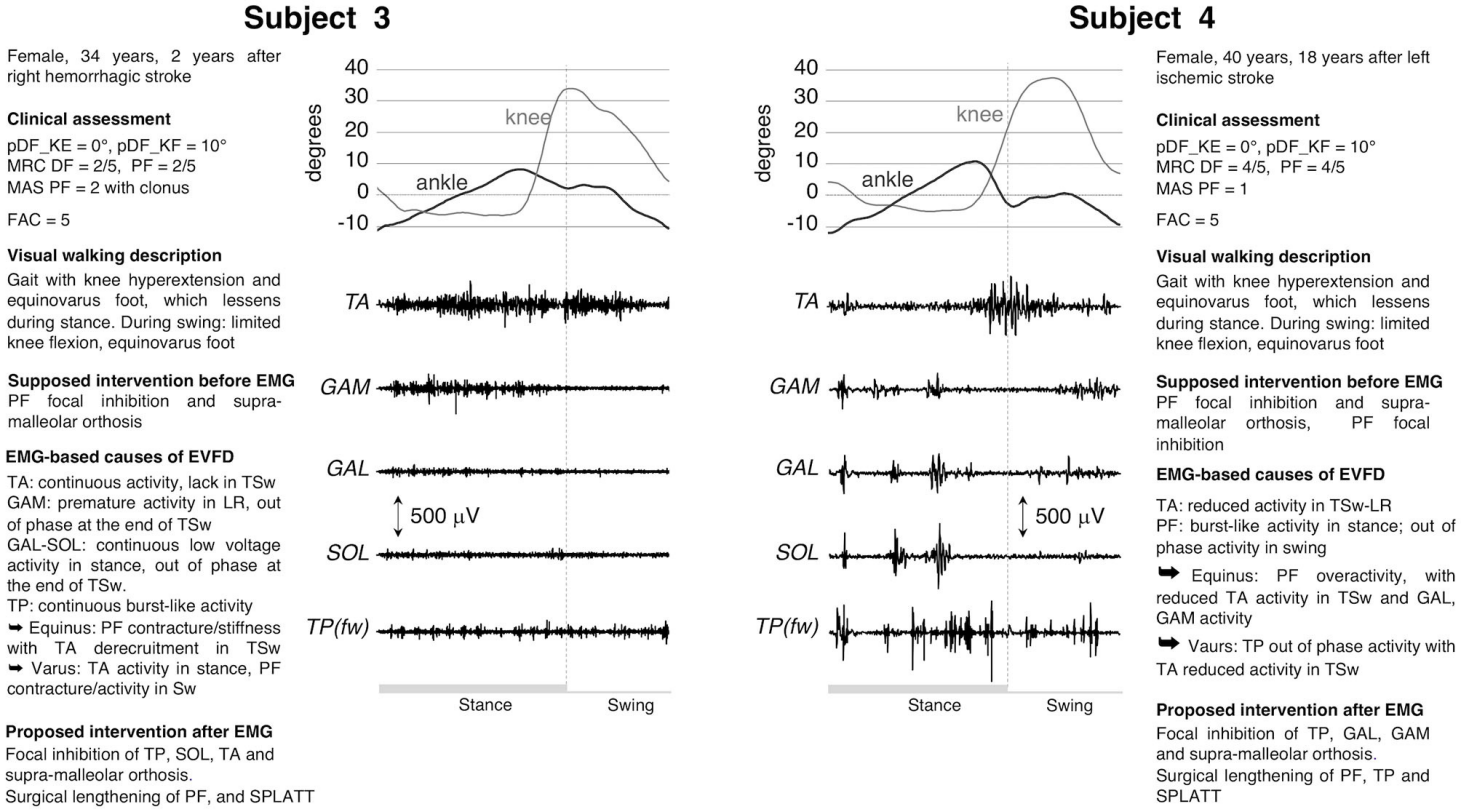
dEMG, knee, and ankle kinematic during a gait cycle for two post-stroke patients presenting similar clinical characteristics and very similar kinematics. See caption in Figure 1 and Table 1 for acronyms. In these two subjects the dEMG of the tibialis posterior muscle was recorded using fine wire electrodes [TP(fw)].

Subjects #3 and #4 (Figure 2) had a gait with EVFD, with reducible equinus during stance, a hyperextended knee in MSt-TSt and a knee flexion deficit in swing. Clinically, both cases exhibited slightly reduced passive ankle mobility, which is consistent with a modest shortening of PF. In Patient 3, there was PF spasticity and muscle force reduction of both PF and DF. Following a clinical evaluation, the treatment suggested was the use of orthosis (to compensate for the DF deficit in swing and to contain the varus attitude) and a generic focal inhibition of PF muscles. The dEMG assessment showed completely different dEMG patterns for the two subjects, which suggested modifications of interventions (Figure 2). The EMG morphology seen in Patient 3 is typical when an increase in PF stiffness is present. Because of this passive resistance, during swing TA failed to lift the foot and decreased its activity in terminal swing (TSw). The continuous activity of TA could explain the varus during stance and contributed to the varus during swing, along with the contracture/stiffness of SOL. Therefore, in this subject, the focal inhibition was only aimed at correcting the varus component determined by a combination of SOL, TP, and TA. In addition to this, there is a surgical possibility of lengthening the whole PF muscle-tendon unit and of performing a split anterior tibialis tendon transfer procedure (SPLATT) to provide a balanced foot dorsiflexion, the latter being supported by the appropriate activation of the TA muscle. In Patient 4, the lack of dorsiflexion during swing was caused by a combination of out-of-phase activity of PF and reduced activity of TA in TSw. In this patient the orthosis and the focal inhibition of the GAM, GAL and TP muscles, but not the SOL, were confirmed. In addition, the surgical lengthening of the PF, TP muscles and SPLATT could be a viable option.

**Figure 3:**
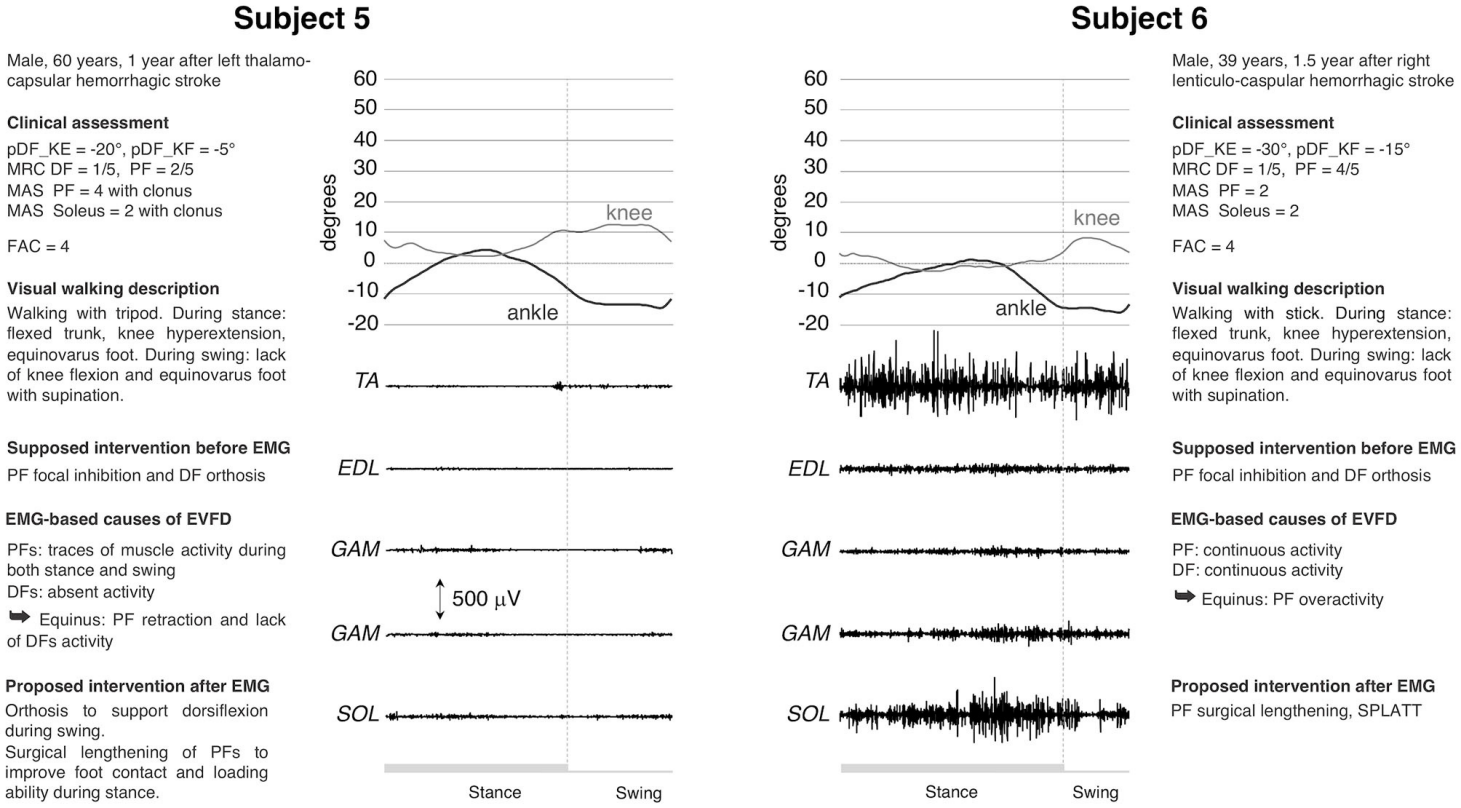
dEMG, knee and ankle kinematic during a gait cycle for two post-stroke patients presenting similar clinical characteristics and very similar kinematics. See caption in Figure 1 and Table 1 for acronyms. The completely different muscle activation pattern underlying the same lower limb kinematic is evident.

Subjects #5 and #6 (Figure 3) exhibited EVFD during stance and supination during swing, knee extended during the entire stance phase and knee flexion deficit during swing. Clinically both patients had the same functional ability and a significantly reduced passive ankle mobility that is compatible with PF retraction. In both cases there was a marked weakness of TA and a spasticity of PF, the latter was more pronounced in Patient 5, who also has less residual strength and clones at the bedside assessment (see Figure 3). Following the clinical evaluation, the treatment suggested was the use of orthosis and the possible focal inhibition of the PF muscles. The dEMG of these patients showed two completely different patterns. In Patient 5, the total absence of DF and PF activities led to favor surgical lengthening of PF, rather than opting for focal inhibition. In this case, surgery would be aimed only to restore a proper foot placement on the ground. In Patient 6 the continuous activity of TA was counteracted by the premature and out-of-phase activity of PF in LR and swing, respectively. The continuous activity of TA could explain the varus component during stance and contributed to the varus in the swing phase along with the activity of SOL and the lack of EDL activity. The pulling action of EDL, in normal subjects, compensates the varus component of TA during dorsiflexion. The presence of continuous PF activity during walking (see Figure 3) confirmed the appropriateness of focal inhibition to all PF muscles and advocated for an intervention to lengthening them. The presence of important TA activity without EDL supported the SPLATT procedure.

The use of dEMG also addresses the physiotherapy aspect of a treatment. For example, in cases 1, 2, and 4, TA had a proper recruitment of muscle motor units (as proven by interferential signal) that allowed for foot dorsiflexion. This was missing in TSw. Therefore, TA failed either to overcome the tension of the contracted and hyperactive PFs (cases 1 and 4) or the stiffness (cases 2 and 3). Physiotherapy should increase the ankle range of motion for all patients, the tolerance to PF lengthening in cases 1 and 4 and augment the strength during lengthening in cases 2 and 3. Any strengthening of the dorsiflexors would be meaningful only after PF recovers proper extensibility, without hindering passive movements and without responding to stretching maneuvers during gait.

Even for case 6, TA did not require treatment: MRC score was only 1/5 at the bedside evaluation, but EMG signal was properly represented during walking (correct recruitment and derecruitment of many motor units leading to an interferential signal), thus enabling foot dorsiflexion. In case 5, physiotherapy would not be appropriate given the substantial absence of muscle recruitment in the leg muscles.

## Discussions

The limited availability of literature regarding the impact of dEMG on the choice of the rehabilitative treatments and on the subsequent patient outcomes is probably the main barrier to its systematic use. To start addressing this issue, we presented data from six patients in which the added value provided by dEMG was evident. In 5 out of 6 cases, the suggested treatment was revised following dEMG assessment.

dEMG enables professionals to understand what the primary cause of EVFD is, showing what combination of paresis, activation, overactivity, stiffness, contracture/shortening is present in a specific patient. In all the patients examined, three aspects can be emphasized:

- Despite clear similarities in their main impairment, observational gait assessment, and joint kinematic, relevant differences in muscular behavior were present (13);

- EMG assessment led to a change in the therapeutic treatment with respect to focal muscle inhibition, also favoring targeted surgical proposals. This aspect has already been reported by other authors (6, 7, 26–28);

- Physiotherapy treatments can benefit from the information provided by dEMG. Timing, amplitude and morphology of the dEMG signal can shed light on the residual level of motor control, muscle recruitment, interference of both muscle overactivity and non-neural components (6). Actually, knowing the causes for an alteration in the gait pattern of a patient, allows to set specific targets tailored to the patients' impairment, integrating the predefined physiotherapy protocols that are often used in everyday practice (29). Tailored physiotherapy should include intensive motor training, daily stretching at high load and exercises characterized by maximal amplitude rapid alternating movements (30).

Our comments on the added value provided by dEMG in the assessment and treatment selection in neurological patient are in line with the results of similar studies, where dEMG was used to quantify the different forms of muscle overactivity, including spasticity (7, 31–33) and to support the design of neuro-orthopedic surgery (27, 28). A thorough review of the applications of sEMG in neurologic rehabilitation can be found in a study by Campanini et al. (13).

### How to Use dEMG in the Choice of EFD and EVFD Treatments

The numerous combinations of paresis, muscle shortening, imbalance and overactivity that might result in EFD or EVFD have been summarized in Table 1. Generally speaking, the presence of PF overactivity and/or of bursts-like activity during stance and out-of-phase activity during swing promotes focal inhibition. Moreover, dEMG reveals which muscles are specifically involved. In addition, the characteristics of the dEMG signal (i.e., timing, amplitude, and morphology) could provide useful information when choosing the type of inhibitory pharmacological treatment (nerve block or inoculation of botulinum toxin). The absence of EMG activity and/or the presence of a very reduced and continuous activity, excludes the usefulness of focal inhibitions. In this case the main cause of EVFD is PF muscle shortening and/or increased stiffness. The presence of preserved, or partially preserved recruitment of PF muscles during stance supports the choice of surgical lengthening. In this case the propulsion provided by PF muscles should result in an improvement of functional walking (speed, fluidly, and energy cost) in addition to the mere correction of the foot. The absence of DF activity indicates the need for an orthosis, and this need will remain even after PF lengthening. Moreover, in functional surgery, the presence of TA interferential signal during swing is a pre-requisite for DF tendon transfer (as in the case of Subject 6).

From the physiotherapist's point of view, GA and sEMG can provide optimal treatment protocol (34). The type of signal recorded on DF and PF can indicate whether and how to treat these muscle groups in the presence of EVFD. When the TA signal is interferential and phasic, the recovery of PF length should be targeted. If PFs have a bursts-like signal, the patient could benefit from a treatment to recover muscle extensibility and decrease linear resistance caused by an increase in transverse collagen fibers and a decrease of the slide between muscle and fascia, respectively (35). This could be achieved by stretching exercises and exercises characterized by maximal amplitude rapid alternating movements to prevent contracture, to improve joint range of motion and flexibility and by muscle strengthening (30, 36–43). Soft tissue manual treatments could be helpful too (35, 44).

### Barriers to the Use of dEMG in Clinical Practice

The void between the clinical usefulness of a dEMG assessment in patients with EVFD and its limited use in the clinical and physiotherapy fields led us to share the cases presented in this work. While the added value of dEMG, when working with neurologic patients is confirmed among experienced users (45), it is not known to most professionals (16). This is reasonably due to the scarcity of clinical literature, and especially of clinical trials, which could demonstrate greater benefits for patients when the treatments are selected by integrating clinical assessment with dEMG (46).

A further barrier to the use of dEMG lies in the difficulty of properly performing and interpreting the results. The analysis of the dEMG signal (15), its interpretation from the physiopathological point of view, the relationship between an altered signal and an observed deviation, and the choice of therapeutic treatment in light of the instrumental data require specific knowledge. In addition, technical skills are necessary, such as the ability to recognize (and not comment on) artifacts and the knowledge that the effects of technical variables (e.g., gain, filters, etc.) has on data (15). Indwelling EMG requires for a time-consuming prepping and provides data that can be corrupted by large motion artifacts. All these steps require time, teamwork, in depth knowledge of physiopathology, and an appropriate setting. In our opinion, the skills necessary to conduct and interpret a dEMG examination and the availability of the necessary equipment are not suited for the daily practice. However, these are available at a motion analysis labs (MAL), where specialized staff received specific training and over the years collected hundreds or thousands of case data. These structures, where available, represent a valuable asset for other rehabilitative services in the local area. Since the learning curve to carry out and interpret an exam correctly takes years, it seems crucial that the operators involved in a MAL should be the one to handle this topic. The selection of the teaching staff, inclusive of physiotherapists, motor scientists, medical specialists in rehabilitative medicine, neurologists, and biomedical engineers, is also fundamental. At least 5 years of practical experience with dEMG techniques are suggested to qualify in order to be able to teach and train dEMG to clinical neurorehabilitation professionals (45).

Technical and cultural barriers could be overcome by enriching the university curricula to include rehabilitation classes and lectures by the professionals who work with walking biomechanics and instrumental evaluation (13). In addition, further efforts are needed to develop courses on how to interpret dEMG signal, leading to a greater consistency of data interpretation among the different centers.

### Limitations

This manuscript is part of a Special Topic relating to “Surface Electromyography: Barriers Limiting Widespread Use of sEMG in Clinical Assessment and Neurorehabilitation.” Therefore, it focuses on the added value of sEMG without providing details on the complete clinical assessment of patients, on the individual's pathophysiology of spasticity, on the functional assessment of walking by means of clinical scales, and on gait biomechanics as provided by GA (47). Similarly, it does not compare the effectiveness of treatments when designed based either on the sole clinical evaluation or with the contribution of dEMG.

## Conclusions

This paper is a presentation of anecdotal cases that show the usefulness of dEMG during clinical decision-making and physiotherapy. Both technical and knowledge-related barriers determine its current limited used in the clinical practice. Adequate training during university, further literature on the topic, along with strategic communication and reliable opinion leaders, are needed to overcome these barriers.

## Data Availability Statement

Inquiries can be directed to the corresponding author/s.

## Ethics Statement

Ethical review and approval was not required for this educational paper on human participants in accordance with the local legislation and institutional requirements. Written informed consent to clinical and instrumental data publication was provided by all subjects.

## Author Contributions

IC: concept and design, study coordination, selection of the cases, interpretation of data, preparation of manuscript, and review of manuscript. MC: concept and design, selection of the cases, interpretation of data, and review of manuscript. MM: concept and design, interpretation of data, and preparation of manuscript. AM: concept and design, preparation of manuscript, and review of manuscript. All authors contributed to the article and approved the submitted version.

## Conflict of Interest

The authors declare that the research was conducted in the absence of any commercial or financial relationships that could be construed as a potential conflict of interest.
